# The Cost-Effectiveness of Using Epoetin-Beta Versus Darbepoetin-Alfa for the Treatment of Anemia Among Chronic Hemodialysis Patients

**DOI:** 10.7759/cureus.11895

**Published:** 2020-12-04

**Authors:** Hanan AlKharboush, Fatimah Alshehri, Ibrahim Alatwi, Khaled Al Karni, Abdurahman Alatawi, Ahmed M Hamdan

**Affiliations:** 1 Pharm D Program, Faculty of Pharmacy, University of Tabuk, Tabuk, SAU; 2 Pharmaceutical Care Administration, General Directorate of Health Affairs in Tabuk Region, Ministry of Health, Saudi Arabia, Tabuk, SAU; 3 Department of Pharmaceutical Care, King Khaled Civil Hospital, Tabuk, SAU; 4 Pharmacy Practice, Faculty of Pharmacy, University of Tabuk, Tabuk, SAU

**Keywords:** darbepoetin-alfa, epoetin-beta, chronic kidney disease, anemia, cost effective analysis

## Abstract

Background and objective

Anemia is a common prognosis of chronic kidney disease (CKD). It is predominantly managed with synthetic erythropoietin. The principal objective of this study was to compare the cost-effectiveness of the use of short-acting erythropoietin with the long-acting one to maintain serum hemoglobin (Hb) concentration within the range of 10.5-12 g/dL.

Method

This was a retrospective cohort study involving patients diagnosed with stage 5 CKD according to the Saudi Society of Nephrology and Transplantation conducted at eight tertiary care centers in the Tabuk region, Saudi Arabia. We compared the cost-effectiveness of long-acting erythropoietin with the short-acting one. The decision analysis model and Markov model were established to simulate a cohort of 55-year-old patients to estimate the incremental cost and quality-adjusted life-year (QALY) for chronic hemodialysis patients (CHP) treated with either darbepoetin-alfa or epoetin-beta for at least nine months. The incremental cost per QALY was the main outcome marker for using both medications. Serum HB levels were monitored on a monthly basis and costs were calculated.

Results

A total of 291 CHP met our inclusion criteria; 194 of them were treated with darbepoetin-alfa while 97 were treated with epoetin-beta. The mean age was 56.3 ± 11.2 years for the darbepoetin-alfa group and 55.2 ± 7.8 years for the epoetin-beta cohort. The baseline serum Hb was 10.68 ± 0.98 g/dL for darbepoetin-alfa patients and 11.63 ± 0.32 g/dL for the epoetin-beta group (p=0.003). We observed a significant difference between the percentage of patients successfully treated with epoetin-beta and those managed with darbepoetin-alfa (80.4% vs. 63.92%, p=0.01) with considerably less cardiovascular side effects. The average annual cost per patient was estimated at $919.47 and $12,319.41 for epoetin-beta and darbepoetin-alfa respectively. Also, the average effectiveness was 0.58 for darbepoetin-alfa vs. 0.61 for epoetin-beta. The average cost-effectiveness ratio was $980.25 and $15,023.66 with an incremental cost difference of -$966 in favor of epoetin-beta compared to darbepoetin-alfa.

Conclusion

Based on our findings, treating anemia in hemodialysis patients using epoetin-beta is very cost-effective compared to managing them with darbepoetin-alfa.

## Introduction

Chronic kidney disease (CKD) has become a focal point in the debate about improving public health all over the world. Globally, 1.4 million patients receive renal replacement treatment with more than one million patients dying yearly from end-stage renal disease (ESRD) [1]. Lately, ESRD has been found associated with a several-fold increase in mortality and morbidity, especially pertaining to the risk of cardiovascular diseases. Today, ESRD is regarded as a public health issue, and it imposes great human suffering and puts a catastrophic economic burden, mainly on poor countries, where less than 2% of the ESRD patients have access to renal replacement therapy. Thus, this leads to premature deaths, particularly among those diagnosed with ESRD at the age of less than 20 years [2]. Moreover, an increase in ESRD rates in the future has been predicted, where it will be more prominent in low-income countries, especially those with a growing population of people with diabetes and hypertension.

Healthcare system planning requires careful assessment of patients with chronic diseases. The epidemiology and treatment of CKD need a careful study regarding its morbidity and mortality rates. The incidence of this disease is on the rise globally [1]. It has been estimated that the global prevalence of CKD is between 11-13% [2]. These patients suffer from renal anemia, especially in the end stages of the disease, since their kidneys cannot secrete enough erythropoietin to produce red blood cells (RBCs) [3]. Protocols of treatment include RBC transfusion (RBCT) or erythropoietin-stimulating agents (ESA) such as epoetin-alfa, epoetin-beta, and darbepoetin-alfa [4]. ESAs are a group of medications that are directed towards bone marrow in order to stimulate the differentiation of progenitor cells to RBCs in the treatment of anemia associated with end-stage CKD. Every ESA requires continuous administration to keep stable serum hemoglobin (Hb) levels in the desired range. In 2017, it was reported that there are between 2,201,128 to 2,595,630 patients in Saudi Arabia with a mortality rate of nearly 9.9% [1].

The risk factors for the incidence of CKD in Saudi Arabia are highly diverse and the nature of the risk is very high. They include hypertension, diabetes, and obesity [5], which are prevalent in Saudi Arabia, especially in the northwestern area: the Tabuk region. The Tabuk region consists of five governorates including approximately 170 villages. According to the official records, the population in Tabuk is estimated to be around one million [6]. There are eight hemodialysis centers with varying capacities in the region; seven are affiliated with the Ministry of Health and one is under private management (DaVita Center). Both RBCT and ESA are used for treating CKD in these centers. It has been reported that using ESA leads to greater improvement in the quality of life (QOL) and quality-adjusted life-year (QALY) compared with RBCT [7-9]. Hence, ESA has become the standard option for treating CKD. ESA class includes short-acting medications such as epoetin-beta and long-acting ones such as darbepoetin-alfa. The principal objective of this study is to compare the cost-effectiveness of these two medications (epoetin beta and darbepoetin-alfa) for managing anemia associated with CKD. Previous health economic studies mainly focused on short-acting medications [10-14]. Moreover, the health outcomes comparing short- and long-acting ESAs and their effects on cost have not been extensively investigated yet in the Tabuk region. In light of this, we performed a retrospective cohort study in order to do a cost-effectiveness analysis from the payer perspective to estimate the potential impact of using two treatment strategies for anemia associated with CKD: short-acting erythropoietin: epoetin-beta three times weekly vs. long-acting erythropoietin: darbepoetin-alfa once monthly. We believe this study can serve as a guide for decision-makers, especially those at the Saudi Society of Nephrology and Transplantation, for devising treatment protocols for chronic hemodialysis patients (CHP).

## Materials and methods

Study design

We selected patient files of patients admitted to the hemodialysis units and diagnosed with CDK stage 5 with anemia from the hospitals' clinical data registry entry from eight tertiary care hospitals in the Tabuk region: Tabuk city (King Khaled Civil Hospital, DaVita Dialysis Center), Haqel, Diba, Alwajh, Albada, Tayma, and Umluj; these admissions spanned a period of one year from January to December 2019. The collected data included the prescribed medications with their doses along with therapeutic outcomes in terms of the monthly follow-up change in serum Hb levels to reach the therapeutic targeted level (10.5-12 g/dL) and the reported side effects that emerged during the course of treatment (cardiovascular side effects). Afterward, we applied the decision analysis model and Markov model to simulate a cohort of 55-year-old patients to estimate the incremental cost and QALY for CHP treated with either darbepoetin-alfa or epoetin-beta for at least nine months.

The inclusion criteria

The inclusion criteria were as follows: all CHP diagnosed with stage 5 CKD (both Saudi and non-Saudi residents in the Tabuk region only; not transferred from other kidney care units outside the Tabuk region) who were above 18 years old with CKD on hemodialysis therapy for at least nine months with the same mode of dialysis and same dosing interval for at least three months. Patients in all centers except DaVita had to be on darbepoetin-alfa (50 or 100 mcg/0.5 mL) once monthly. DaVita Center was using epoetin-beta 1,000 IU thrice weekly. These choices had already been decided by the policies of the healthcare payers, who were the providers of ESA for all patients, and in accordance with physician directions.

We used the laboratory parameters measured on a monthly basis: Kt/V, serum Hb concentration, serum transferrin, transferrin saturation (TSAT), RBC count, hematocrit, platelet count, ferritin, serum intact parathyroid hormone (iPTH), albumin, phosphate concentration, and calcium concentration to follow up the patients. We used a serum Hb level between 10.5-12 g/dL as the target for our cost-effectiveness analysis.

The exclusion criteria

We excluded patients who had received organ transplantation, those who did not complete at least nine months of continuous same therapy, and those who had become anemic due to reasons other than CKD.

Ethical considerations

All procedures were performed in accordance with the ethical standards of the institutional and national research ethics committee (no: UT-99-03-2020) from the University of Tabuk and (TU-077/019/015) 52987-1441 from the Research and Study Center at the Saudi Ministry of Health.

The cost-effective analysis

**Figure 1:**
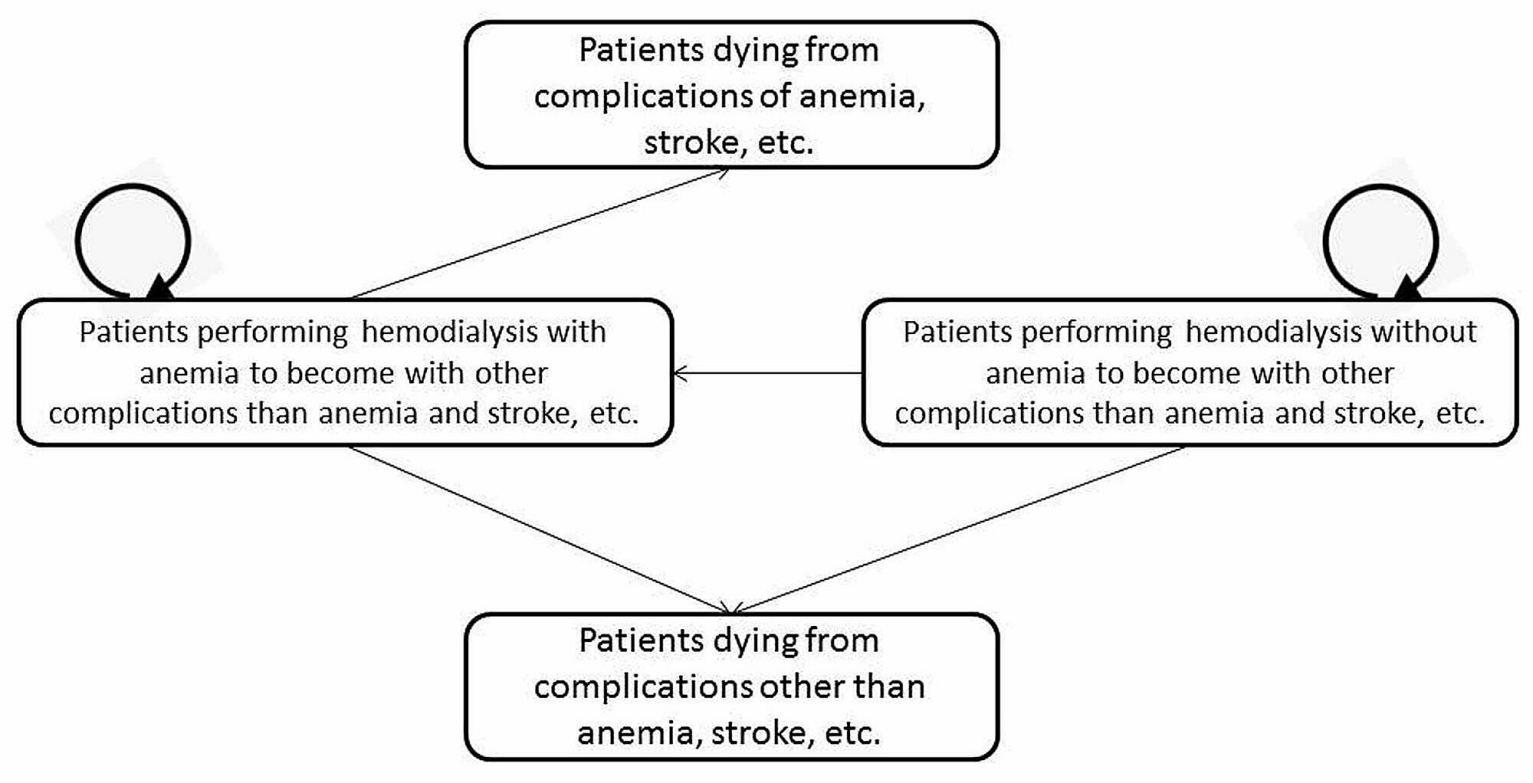
Schematic presentation of the Markov model for the treatment of ESRD patients with erythropoietin ESRD: end-stage renal disease

We used the data for analysis according to the healthcare payer perspective. Moreover, we used the decision-analytic technique to model this situation and Markov's model for evaluation of the average incremental cost of the two medications to treat anemia. The Markov model consisted of four health conditions for each patient, as shown in Figure 1, according to the serum Hb levels; we then compared each condition with a serum Hb level of less than 9 g/dL.

Firstly, for patients who were alive and undergoing hemodialysis and suffering from anemia, stroke, etc., we assigned a serum Hb level of 9-10 g/dL. Secondly, for patients who were alive and undergoing hemodialysis and suffering from any other problem such as catheter infection, we assigned a serum Hb level of 10-12 g/dL. Thirdly, for patients who were dying and suffering from complications of anemia, stroke, etc., we assigned a serum Hb level of more than 13 g/dL. Finally, for patients who were dying and suffering from other complications, we assigned a serum Hb level of 12-13 g/dL. This Markov model had a cycle with a length of four weeks and a lifetime horizon. We used the four-weekly measured targeted serum Hb concentration between 10.5-12 g/dL as the target for both treatments. Those with Hb levels higher than 12 g/dL or lower than 10.5 g/dL were considered as one group and called "patients out of targeted serum Hb." Clinical success rate (CSR) is the proportion of the population achieving the targeted serum Hb concentration. We assumed that the patients would continue to perform hemodialysis until their death. The movement between each health state was assumed to be dependent on the serum Hb level. They were the probabilities that determined the transition between each health status. Costs and QALYs gained were calculated as patients went through this model.

QALY calculation

Calculation of QALYs was performed using the additional number of years of life gained from an intervention multiplied by a utility judgment of the QOL. The QALYs gained can be calculated using the probabilities to determine the mean, variance, and probability distribution for the QALYs gained. The Markov model was established to simulate a cohort of 55-year-old people. It was carried out using TreeAge Pro 2020 (TreeAge Software, Inc., Williamstown, MA).

The cost elements in Saudi Riyals in reports for every month were converted to US Dollars ($) (one US Dollar equals 3.77 Saudi Riyal). We applied the Markov model of a hypothetical cohort of dialysis patients treated with epoetin-beta three times weekly or darbepoetin-alfa once monthly for one year. We applied the decision-analytical technique using TreeAge Pro 2020.

Assumptions

For model inputs, we assumed that there was no change related to hospitalizations between the two treatments (no extra professional visits, advice, oxidative stress, etc.), no difference between surveillance costs between the two treatments, and no difference between the two groups of patients regarding hospital visits for hemodialysis irrespective of the treatment type. Moreover, we assumed that the laboratory assays were the same in all hemodialysis centers. Also, we assumed that there was no difference between the serum Hb target levels between male and female patients. We also assumed that there was no significant dose difference between the two medications given to patients with central nervous catheters and those taking them using arteriovenous fistula.

Sensitivity analysis and Monte Carlo simulation

Our model included assumptions and uncertainty. Hence, we performed a one-way sensitivity analysis for the individual variation and multi-way sensitivity analysis variation of multi-components at the same time, by varying baseline estimation among the ranges of potentially reasonable values such as a number of patients in the hospitals, the length of stay in hospitals, survival estimates, ESA costs, utilities, etc. Moreover, a probabilistic sensitivity analysis was conducted by a Monte Carlo simulation (MCS) to monitor and to eliminate the uncertainty by creating 50,000 samples. Afterward, we calculated the expected value. Using that model, the expected value was calculated to test and to evaluate whether these changes modified the reached conclusion. Normal distribution of clinical parameters was employed for the relative and baseline risk. A log-normal distribution was applied to the cost estimates.

Statistical analysis

Statistical analyses were done and groups were compared using SPSS Statistics software 24.0 (IBM, Armonk, NY). Depending on the variable distributions between the two groups, we used the Mann-Whitney test or Wilcoxon test. All results of non-normally distributed variables were presented as percentages of discrete variables. A p-value of <0.05 was considered statistically significant.

## Results

Patient cohort characteristics

We initially screened 341 patients in total; 291 CHP met our inclusion criteria and were deemed eligible for analysis (85.34%). Among them, 194 CHP were treated with darbepoetin-alfa, with a mean age of 56.3 ± 11.2 years, and 97 CHP were treated with epoetin-beta, with a mean age of 55.2 ± 7.8 years (Table 1).

**Table 1:** Demographic characteristics and laboratory results of each group of patients treated with short- and long-acting erythropoietin *Statistical significance at a p-value of <0.05 CHP: chronic hemodialysis patients; SD: standard deviation

Variables	Darbepoetin-alfa	Epoetin-beta	P-value*
Number of CHP	194	97	
Age in years, mean ± SD	56.3 ± 11.2	55.2 ± 7.8	0.53
Male, n (%)	99 (51%)	50 (51.55%)	0.69
Primary renal disease, n (%)	62 (32%)	19 (19.6%)	0.01
Diabetes, n (%)	44 (22.7%)	32 (32.9%)	0.01
Hypertension, n (%)	29 (14.9%)	19 (19.6%)	0.02
Obesity, n (%)	17 (8.8%)	17 (17.5%)	0.02
Other, n (%)	42 (21.6%)	10 (10.3%)	0.08
Time of dialysis (hours), mean ± SD	410 ± 24.3	391 ± 9.8	0.034
Pre-dialysis blood pressure			
Systolic BP (mmHg), mean ± SD	124 ± 26.9	131 ± 12.9	0.52
Diastolic BP (mmHg), mean ± SD	79 ± 1.3	80 ± 1.9	0.51
Laboratory results			
Hemoglobin (g/dL), mean ± SD	10.68 ± 0.98	11.63 ± 0.32	0.03
Albumin (g/L), mean ± SD	3.9 ± 0.91	3.9 ± 0.89	0.51
Ferritin (ng/mL), mean ± SD	352.91 ± 53	362.12 ± 129	0.63
Calcium (mg/dL), mean ± SD	9.81 ± 0.23	9.8 ± 0.24	0.74
Phosphate (mg/dL), mean ± SD	4.4 ± 1.21	4.2 ± 1.11	0.62
Triglyceride (mg/dL), mean ± SD	151.3 ± 33.2	142 ± 23.1	0.51
Kt/V, mean ± SD	1.42 ± 0.22	1.5 ± 0.1	0.49
Cardiovascular diseases after taking the medication, n (%)	19 (9.79%)	3 (3.09%)	0.005
Vascular access			
Fistula, n (%)	182 (93.8%)	88 (90.7%)	0.3
Catheter, n (%)	12 (6.2%)	9 (9.3%)	0.3

As shown in Table 1, there was no significant difference between the two groups in terms of age, gender, and laboratory tests other than Hb level (p=0.03 for Hb levels). The numbers meant nothing. Meanwhile, there was a higher burden of accompanying diseases such as diabetes, hypertension, and obesity in the group receiving epoetin-beta (p<0.05) (19 patients on darbepoetin-alfa vs. three patients on epoetin-beta; p=0.005). This indicated that patients treated with epoetin-beta were more complicated cases. There was no significant difference between the two groups in the percentage of patients using catheters for hemodialysis. However, there was a significant difference between the two treatment groups in terms of achieving targeted Hb concentration (p=0.03).

Efficacy evaluation

As shown in Table 2, we noticed that the time of dialysis in the epoetin-beta cohort was significantly less than that in the darbepoetin-alfa group: 3.5 hours for darbepoetin-alfa vs. 2.8 hours for epoetin-beta (p=0.034), with a significant difference between the monthly measured baseline Hb levels in the two groups (10.68 ± 0.98 g/dL for darbepoetin-alfa vs. 11.63 ± 0.32 g/dL for epoetin-beta, p=0.001). We noticed significantly reduced cardiovascular problems in patients taking epoetin-beta compared to those taking darbepoetin-alfa (p=0.05). Moreover, we found that a significantly less number of patients with lower HB levels (<10.5 g/dL) or higher Hb levels (>12 g/dL) used epoetin-beta (19.6% and 36.08% for epoetin-beta and darbepoetin-alfa respectively). Hence, the relative risk reduction (RRR) of using epoetin-beta compared to using darbepoetin-alfa was 16.48% for the risk of not obtaining the required Hb range level. The effectiveness was 80.4% and 63.92% for epoetin-beta and darbepoetin-alfa respectively to achieve our target monthly measured Hb levels. So, the cost-effectiveness was $980.25 and $15,023.66 for epoetin-beta and darbepoetin-alfa respectively to treat one patient per month successfully. Hence, the incremental cost was about -$966 per additional case to successfully achieve the required range of monthly measured Hb level.

**Table 2:** Laboratory analysis of patients treated with either epoetin-beta or darbepoetin-alfa *Statistical significance at a p-value of <0.05 Hb: hemoglobin; IV: intravenous; CSR: clinical success rate; SD: standard deviation

Parameter	Darbepoetin-alfa	Epoetin-beta	P-value*
Number of patients	194	97	
Hb level (g/dL), mean ± SD	10.68 ± 0.98	11.63 ± 0.32	0.03
Transferrin saturation (%)	26.9	29.2	0.04
Ferritin (ng/mL)	287.9	298.6	0.42
IV iron (%)	83.9	88.9	0.59
IV iron dosage (mg/month), mean ± SD	110.3 ± 11.3	115.9 ± 19.1	0.51
Drug dosage, mean ± SD	5,980 ± 2,987 IU	98.4 ± 39.8 µg	
CSR, n (%)	124 (63.92%)	78 (80.4%)	0.02
Patients with Hb of >12 g/dL or <10.5 g/dL, n (%)	70 (36.08%)	19 (19.6%)	0.01
Dialysis time per patient per session (hours)	3.5	2.8	0.06
Cardiovascular diseases after taking the medication, n (%)	19 (9.79%)	3 (3.09%)	0.05

Baseline clinical costs and input assumptions

**Figure 2:**
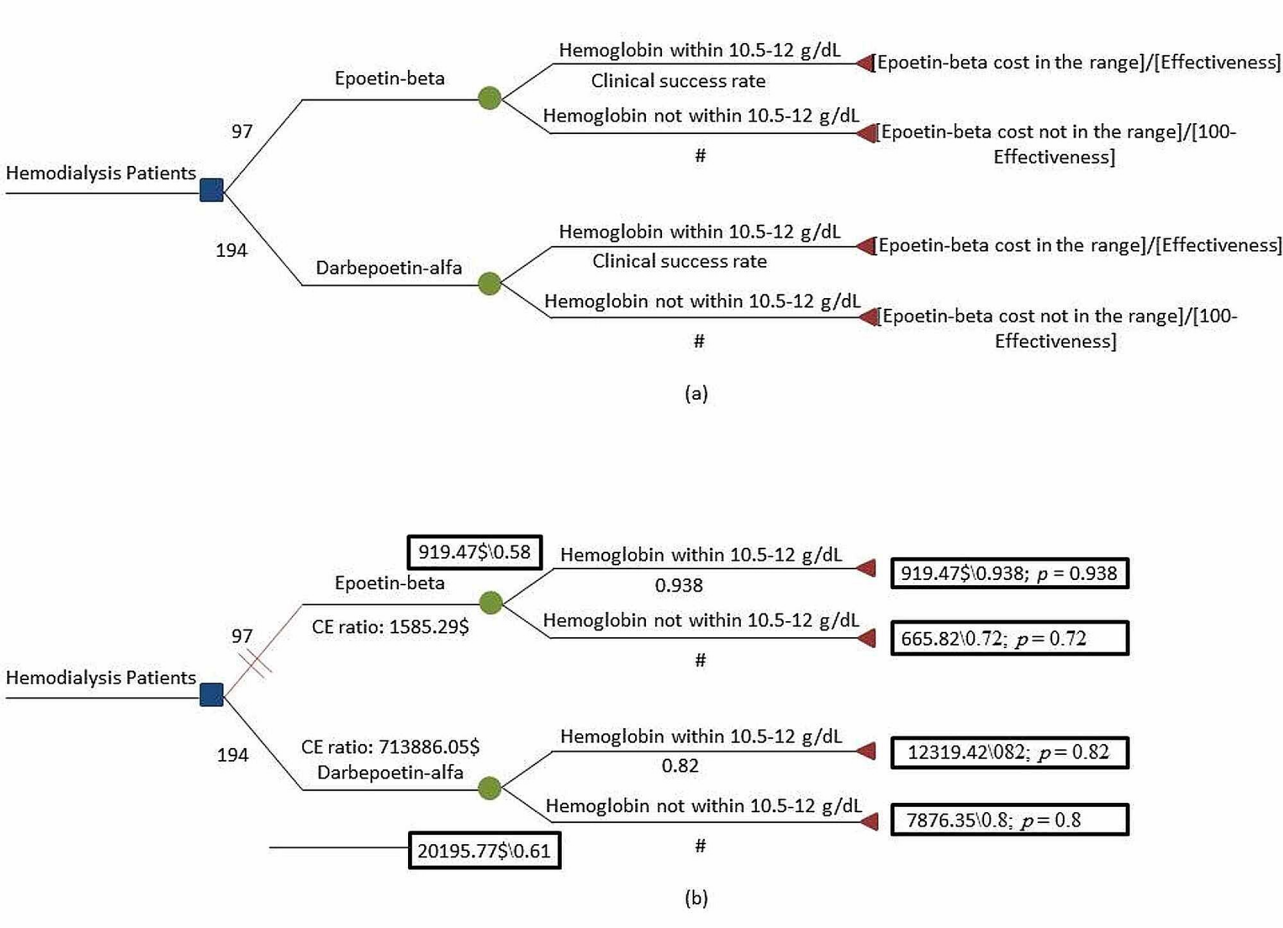
Cost-effectiveness analysis of epoetin-beta vs darbepoetin-alfa in the treatment of anemia associated with CKD in hemodialysis patients (a) Decision tree framework. (b) Results of cost-effectiveness analysis after roll-back calculation CKD: chronic kidney disease

As shown in Table 3, over a time horizon of one year, we considered the direct medical costs for both epoetin-beta and darbepoetin-alfa including fees for medical consultant, medical specialist, nutritional specialist, pharmacist, and social specialist, and the cost incurred for iron usage, medication usage, and hospitalization. We assumed that there was no significant difference in terms of hospitalization and the length of stay for both medications. The average annual cost per patient was estimated at $919.47 and $12,319.41 for epoetin-beta and darbepoetin-alfa respectively. There was no statistically significant difference between using the two medications in the monthly monitored parameters, as shown in Table 1. The cost-saving associated with using epoetin-beta was $11,399.95. The average annual effectiveness estimated using our model was 0.58 and 0.61 for darbepoetin-alfa and epoetin-beta respectively. So, the average cost-effectiveness was $533.29 and $713,886.05 for epoetin-beta and darbepoetin-alfa respectively (Figure 2).

**Table 3:** Baseline clinical costs per month and input assumptions

Variables	Base-case estimate per patient per year ($)
Medical consultant	3,405.6
Medical specialist	2,311.2
Nutritional specialist	1,542
Pharmacist	1,455.6
Social specialist	1,180.8
Darbepoetin-alfa cost	6,638.1
Epoetin-beta cost	915.2
Hospitalization for darbepoetin-alfa	19,050.759
Hospitalization for epoetin-beta	19,155.7984

**Figure 3:**
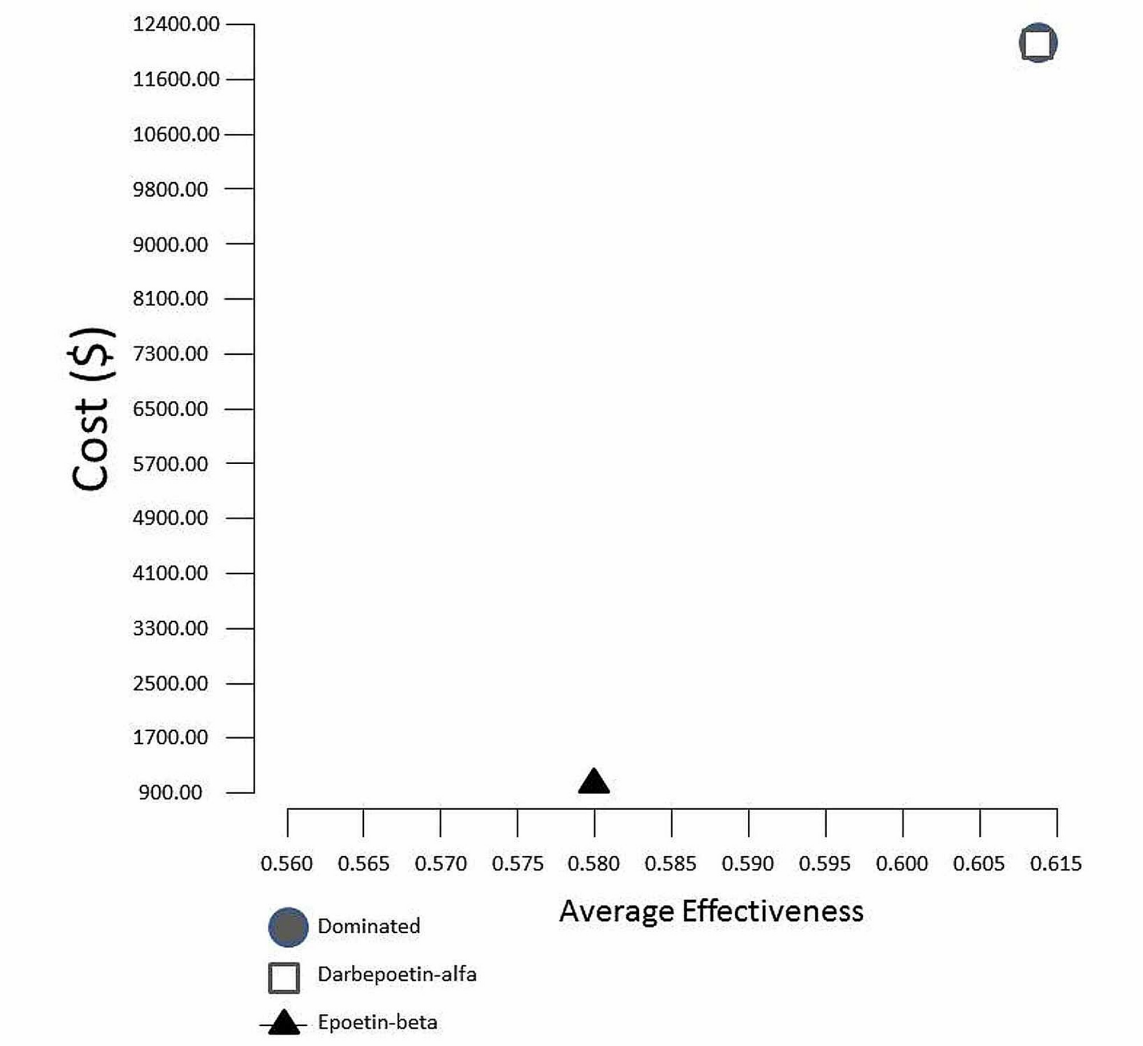
Cost-effectiveness diagram Epoetin-beta dominated darbepoetin-alfa since it is highly effective and less costly

Additionally, the treatment of anemia associated with hemodialysis CKD patients with epoetin-beta dominated darbepoetin-alfa as shown in the cost-effectiveness diagram (Figure 3).

Quality-adjusted life-year

QALY is a tool used for evaluating the health benefits of using medications. So, we used QALY analysis to examine preferential therapeutic outcomes of using one medication over the other. Moreover, we calculated the incremental cost per additional QALY. In Table 4, cost, QALYs, and incremental cost over a one-year time horizon associated with epoetin-beta dominating darbepoetin-alfa administration are summarized. The average annual cost per patient was estimated at $919.47 and $12,319.41 for epoetin-beta and darbepoetin-alfa respectively. In our model, epoetin-beta administration was associated with an increase in patients’ QOL by 0.1 QALYs per additional life-years gained on average compared with darbepoetin-alfa over the lifetime horizon with an incremental cost of $12,297.86 for darbepoetin-alfa and $897.92 for epoetin-beta. Therefore, the cost-utility ratio was calculated at 20,845.01 and 1,519.32 $/QALY for darbepoetin-alfa and epoetin-beta respectively, with an incremental cost-utility ratio (ICUR) of darbepoetin-alfa and epoetin-beta at 6,1597.05 and 4,597.35 $/QALY respectively.

**Table 4:** Costs, QALY, and incremental cost per year QALY: quality-adjusted life-year; ICUR: incremental cost-utility ratio

Intervention	Average cost ($)	Incremental cost ($)	Average QALY	Incremental cost	Cost-utility ratio	ICUR ($/QALY)
Darbepoetin-alfa	86,135.1	12,297.86	0.591	1,273	20,845.01	6,1597.05
Epoetin-beta	20,774.3	897.92	0.591	869	1,519.32	4,597.35

For the one-way sensitivity analysis, the inputs in the model were allowed to vary within clinically plausible ranges. Table 4 shows the sensitivity of the base-case ICUR of darbepoetin-alfa and epoetin-beta in different scenarios tested. The model was most sensitive to hospitalization costs and hospital stay, and any increase in those parameters resulted in a decrease in ICURs as reported in Table 4. Assuming utility and survival improvement with darbepoetin-alfa and epoetin-beta use while all costs remained the same resulted in a decrease in ICUR at 61,597.05 $/QALY for darbepoetin-alfa and 4,597.35 $/QALY for epoetin-beta. Testing the hypothesis of equal mortality rate between darbepoetin-alfa and epoetin-beta use resulted in an ICUR at 20,878 $/QALY and 23,940 $/QALY respectively. Decreasing the acquisition cost of darbepoetin-alfa and epoetin-beta by 25% resulted in an ICUR at 11,911 $/QALY and 14,088 $/QALY respectively.

Sensitivity analysis and Monte Carlo simulation

**Figure 4:**
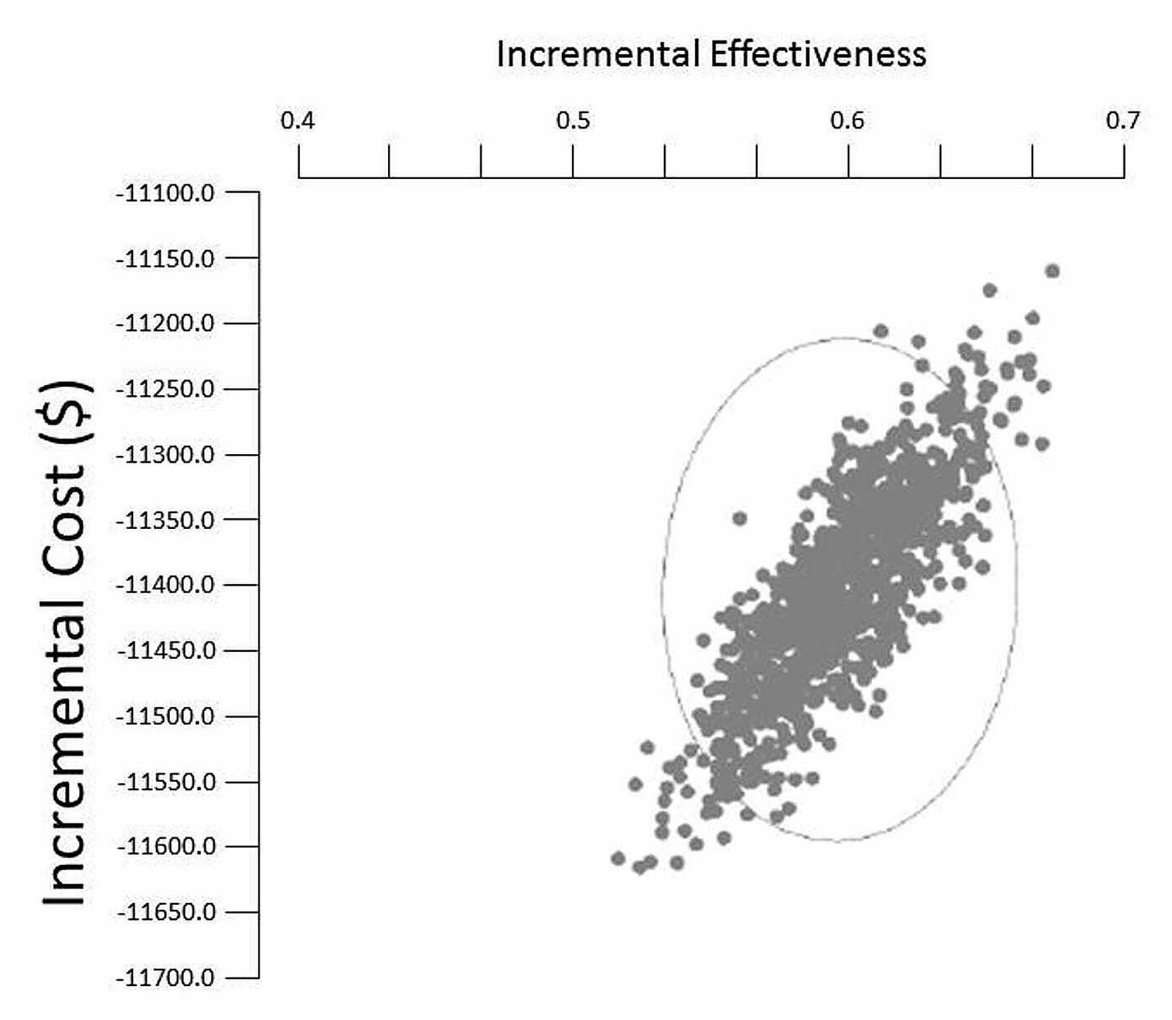
Results of Monte Carlo simulation of incremental cost and effectiveness of scattered plot of epoetin-beta versus darbepoetin-alfa based on 50,000 random iterations of the cost-effectiveness model

We did a one-way and multiway sensitivity study of the results by MCS. For this analysis, 50,000 random simulated trials were run (Figure 4).

**Figure 5:**
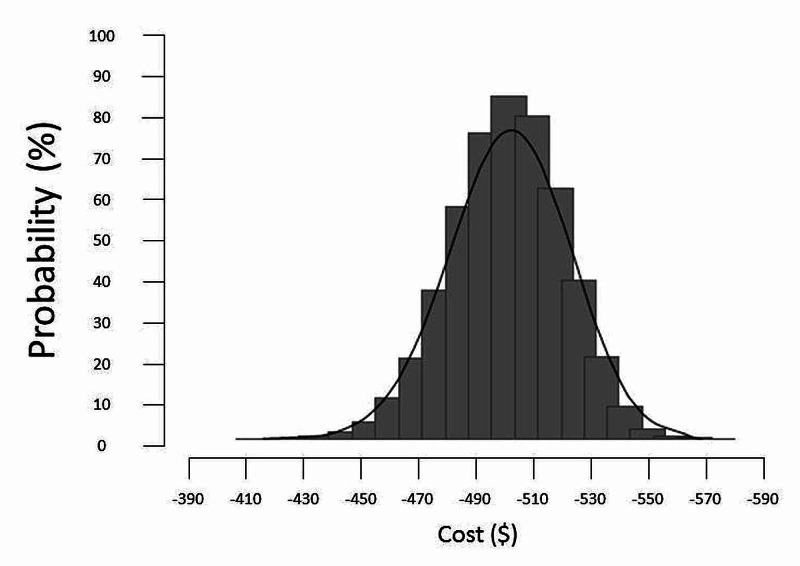
Probabilistic sensitivity analysis distribution of epoetin-beta versus darbepoetin-alfa

The scattered plot showed the robustness of the results; 100% of the results were in the lower right-hand part of the graph (best clinical effect and lower cost). So, epoetin-beta dominated darbepoetin-alfa in the clinical effectiveness parameter with lower cost. Also, the robustness of the calculated ICUR was confirmed by the probabilistic sensitivity analysis distribution of epoetin-beta versus darbepoetin-alfa based on 50,000 random iterations (Figure 5). The analysis showed that the incremental cost between these two medications remained in the range of 10%.

## Discussion

This research involved a comparison of the potential therapeutic and economic impact of using two erythropoietin drugs (epoetin-beta and darbepoetin-alfa) among patients with stage 5 CKD for the treatment of associated anemia. Our pharmacoeconomic analysis showed that anemia in CKD patients treated with epoetin-beta is associated with a substantial clinical benefit and results in significant additional cost savings relative to darbepoetin-alfa. This finding is consistent with that of most studies [10-16]. Elaborated sensitivity analyses revealed that the healthcare payer should be willing to pay at least $11,399.95 (the difference between $12,297.86 for darbepoetin-alfa and $897.92 for epoetin-beta) per additional QALY for darbepoetin-alfa to become cost-effective compared to epoetin-beta. Moreover, the present study found that epoetin-beta is more cost-effective than darbepoetin-alfa, making it the dominant treatment modality for the management of anemia in CHP.

Our data contrast with the findings of Maoujoud et al. [11], who concluded that darbepoetin-alfa is more effective than epoetin-beta in a prospective cohort study of Moroccan patients. These differences in findings may be due to the genetic variation between our patients and the Moroccan cohort. Moreover, the study involved administering epoetin-beta for six months. After that, they shifted patients to darbepoetin-alfa. So, the erythropoietin cycle may need to be longer to get good results. Meanwhile, our data align with that of Schmid [13]. His literature review of studies that dealt with the cost or the cost-effectiveness of darbepoetin-alfa was poor. The main limitation of that study was that he referred to 18 publications in his analysis, and most of the available data was from meeting abstracts (11), and only seven published studies were from peer-reviewed journals. The majority of included studies were retrospectives, and reported data were only about the cost of therapy after a switch to darbepoetin-alfa at a single center. Our study proved that epoetin-beta is superior to darbepoetin-alfa in treating anemia with less cardiovascular side effects based on the data from eight centers of hemodialysis.

## Conclusions

We performed a cost-effectiveness analysis regarding the use of darbepoetin-alfa (long-acting ESA) versus epoetin-beta (short-acting ESA) for the treatment of anemia associated with stage 5 CKD to evaluate their potential outcomes. The findings from our economic model revealed that the use of epoetin-beta was associated with additional cost benefits compared to darbepoetin-alfa. Besides, healthcare payers should be willing to pay at least $11,399.95 per additional QALY for darbepoetin-alfa to become cost-effective compared to epoetin-beta. These findings were robust to the sensitivity analysis. Our study also found that the use of epoetin-beta significantly improves the patients' QOL.
